# Putting the Boot in

**Published:** 1987-05

**Authors:** J. L. Burton

**Affiliations:** Consultant Dermatologist, Bristol Royal Infirmary, Bristol BS2 8HW


### Putting the boot in

**J. L. Burton, B.Sc., M.D., F.R.C.P.**
Consultant Dermatologist, Bristol Royal Infirmary, Bristol BS2 8HW

Two of the happiest days of my life have been ruined by inappropriate footwear. The first time was on my honeymoon. Not in bed, I hasten to add. We Northerners admit to being sewn into our woolly vests from November to March, come what may, but when it comes to amorous dalliance we always remove our boots, and often our socks, weather permitting.

The honeymoon in question was spent in the Austrian Alps, and it was on an alp that the footwear problem first arose. In those days I was a keen rock-climber and on my last holiday as a bachelor I had triumphantly led Tower Ridge on Ben Nevis. Actually I should have been second on that occasion, but my male climbing partner had what doctors call a temporary disturbance of the bowel habit as a result of over-indulgence in the Glencoe rough cider on the previous evening, and it seemed decidedly less risky to lead, even if I did have to climb above my usual standard. It scarcely seemed necessary therefore to take my climbing boots for a gentle wander around an alp or two, especially as I was anticipating frequent pauses for refreshment, flower-picking, photography, amorous dalliance etc. in no particular order (my wife's enthusiasm for 'proper' climbing had never been marked, partly because she seems to be congenitally incapable of bringing her foot up to the level of her ear, and her enthusiasm had dwindled to zero as soon as the engagement ring was safely in place).

Thus it was that on about the fourth day of our honeymoon, we thought we'd try another sport, and decided, boots or no boots, to conquer a peak or so.

Now it's an unfortunate fact that Austria is thickly populated with Fat German Ramblers (FGR), whose interest in food is exceeded only by their interest in clothes. 'Manners makyth man' may be regarded as a bit of a joke in U.K., even by Wykehamists, but in Austria the old German proverb 'Kleider machen Leute' (Clothes make the man) cannot lightly be disregarded. The FGR compete with each other with regard to the size of their paunches and the magnificence of their rambling attire. The former are cultivated by regularly taking a cable car up to around 6000 ft., then walking sedately for 200 yards or so along a level grassy path to a splendid mountain restaurant, where the warmest hours of the day are whiled away in guzzling litres of fizzy beer and sinking huge plates of Bratwurst, Aufschnitt, Apfelstrudel etc., not to mention the irresistible cream cakes. The correct outfit for this type of belly-building expedition consists of a large rucksack, a silver-headed walking stick, expensive lightweight walking boots, red woolly socks, snazzy climbing breeches with decorative braces, red check shirt and a Tyrolean hat festooned with an impressive collection of badges and feathers. A broad belt with a tasteful floral motif holds the whole outfit together, and provides a support for the carefully nourished paunch to flop over.

I may mention in passing that the waitresses in these mountain restaurants are similarly attired in the Austrian national dress, but what they support with their broad belts and built-in Büstenhalter somehow seems that much more attractive. The calculated way they lean over the table to deal with Die Rechnung is certainly not conducive to careful counting of the change from a 100 Schilling note. I quickly learned though, that on honeymoon the correct way to draw the attention of one's companion to these outstanding attractions is to make a tasteful comment such as 'Don't you find their costumes rather flattering, dear?' rather than, 'God, look at the size of those!' which was the phrase I had previously been in the habit of using to my climbing chums. But I digress.

On the day of our alpine expedition we could not afford to take the cable car, but we enjoyed the solitude of our long haul up several thousand feet of stony track through the pine-scented forest. It was only after we passed the cable car station and came close to the restaurant that we began to be troubled by the FGR. As each resplendant group approached, they would start to mutter to each other, and as we drew level they would shake their heads, purse their lips severely, wag their fingers at us and then point at our comfortable brogues, exclaiming 'Schlechte Schuhe, nicht gut, sehr gefährlich'. At first we used to nod and smile brightly in agreement, but this only seemed to increase their agitation. We would then say 'Nein, sie sind gute Schuhe. Ich bin Englander', with appropriate gestures. At this response the animated garden gnomes would become extremely excited and voluble, emitting an angry buzz of incomprehensible German, which would fade into the distance as we walked on. Fifty yards further on the procedure would be repeated, with an identical group of FGR, who would go through the same ritual of muttering, head-shaking, finger-wagging, shoe-pointing and finally angry denunciation of our rambling gear, common-sense, nationality, and morals and parentage too for all we knew. All very tedious.

Twenty years went by, and I was again in the Austrian Alps with another long-legged lass, but this time it was my 16 year old daughter. We were taking a family holiday in Austria because this seemed the only place which came anywhere near to coping with the varied requirements of myself ('not too expensive'), wife ('somewhere warm and relaxing'), 16 year old daughter ('Nepal, but if not, somewhere with big mountains'), 15 year old son ('lots of frankfurters and fashionable clothes shops'), 7 year old daughter ('swimming and toy-shops') and Granny ('interesting shops and not much walking'). Having exhausted most of the above-mentioned attractions on day one, big daughter and I quickly became addicted to the high-level mountain walks which abound in the Zillertal Alps. On this holiday we'd taken our boots and ice-axes, and having conquered a number of the lesser peaks and passes in the first week, we decided to tackle something a little more ambitious. The route we chose started at the mountain village of Ginzling, climbed steeply through the forest to the Max Hütte, then a steady climb up to the head of the Gunggl valley, over the pass in the shadow of the magnificent Zsigmondyspitze, then a traverse around the snow-slopes on the other side to reach the Berliner Hütte, and finally a long walk back down the Zemmgrund to catch the last bus back from Breitlahner (we hoped).

We had no trouble with the early stages. We marched briskly up the steep track through the fir trees, and since there was no cable car, there were few of our fat feathered friends to be seen, and those we did see puffing slowly up towards their beer and sausages at the Max Hütte were soon left far behind. After a short pause at the hut, we started on the serious business of the day as we left the trees behind and made steady progress up towards the snow-line. As the hut dwindled to a small speck below us, the number of climbers diminished, and those we did see were young, tanned, lean and carrying rope, crampons, short skis, ice-screws, the lot. Maybe this is just another example of Teutonic overkill, I thought. Overgrown Boy Scouts.

It was at this stage that we were hailed by the Ancient Mariner, or to be more precise, the Ancient Garnet-Collector. The Gunggl valley is renowned for its garnets, and a few of the hardy locals scour the remote side-valleys looking for gems. This particular prospector came shooting down a slope towards us like a Messerschmitt attacking a Wellington bomber. He had piercing blue eyes, a lean and hungry look, a no-nonsense manner, and an impenetrable Austrian accent, but we soon gathered that he wanted to know where we were going. When we told him our route, he became excited and voluble, just like the FGR, and the gist of his message seemed to be as follows: 'I say, old chap, that's a helluva long climb, which in view of the late snow this year is not without its dangers, and an effete pair of Wallies like you will never make it in less than 12 hours, and you will certainly miss the last bus from Breitlahner'.

Now to be fair to Herr Garnet, we did perhaps look less than competent by his standards. My hat was floppy, powder-blue and very sweat-stained, and this, together with my old football socks and tucked-in gardening trousers didn't exactly make me a Hermann Buhl lookalike. My daughter too failed to impress him. She is always pale and willowy, and on this occasion she was wearing her off-white, long, baggy 'shorts' which are apparently quite fashionable these days if you dress in the 'Obscure' mode in Clifton, but are novel at high levels in the Austrian Alps. She looked like Edith Sitwell playing tennis with a hang-over. How was he to know that she is as strong as a horse, can go for days without food, has endless stamina, always carries the rucksack for both of us, and would be perfectly capable of carrying me too if necessary.

We were accustomed to dividing Austrian 'estimated walking times' by two, e.g. if the sign said 'To Max Hütte, 1½ hours' we reckoned to do it in 45 minutes, and so we were not too alarmed by his estimation of our physical abilities. Indeed, if I'd known the Austrian patois for 'Micturate elsewhere' I'd have been tempted to use it.

Instead I tried to reason with him. Grasping my daughter's biceps as though I was trying to sell him an Aberdeen Angus for breeding purposes, I said 'Sie ist sehr stark...' (She is very strong, much stronger than she looks, etc. etc.) 'No, No' he replied, shaking his head sadly. 'She is only a slip of a girl, and the Zsigmondyspitze is no place for a girl'. 'Not at all' I exclaimed with spirit, 'She habitually carries full churns of milk up Snowdon before breakfast, and only 2 days ago she went up and down the Schönbichlerhorn in 6 hours'.

He was impressed by this, but not as much as he should have been, and when we made to move on, he again became excited, grabbed my arm and made me look through his binoculars at the snow slopes at the head of the valley, 'Schnee, eis, schlecte Schuhe, vvrroompff!', he said, making a 45 degree sliding motion with his flattened hand. 'Ah, schnee, eis, vrumpf', I said. 'Ja, ja, sehr schlecte Schuhe, sehr gefährlich', he said.

Well I had to admit that the steep snow-slopes at the head of the valley did look extremely icy and vrumpf-making, but nevertheless we thanked him for his advice and decided to press on and take a closer look in case we could pick our way up on the ribs of rock. Our hearts were no longer in it though, and as the snow-slopes loomed larger and larger, we began to hear the occasional muffled reverberations of distant avalanches, as the snow was loosened by the midday sun. The thought of what we'd find on the other side of the ridge finally killed our enthusiasm, and we eventually slunk dejectedly back to the Max Hütte, grappling with a few small 'boulder-problems' on the way down, just to pretend that we didn't care.

At the hotel that evening we shared a table with an Oxford don – an odious fellow whose effortless superiority was about as comforting as a blue-bottle up the nostril. 'I'm surprised you had any difficulty with that route' he said plonkingly. 'We did it last week without any bother at all, didn't we Philip?'. I winced and stirred my soup dully. His son smirked, and I fancied I saw small jets of steam emerge from my daughter's ears.

My wife galloped to the rescue, her reactions having for once been preserved by a relaxing day at the poolside. 'John has to be very careful on ice', she said, 'since he had his artificial leg'.

This was originally broadcast in 'Morning Story', BBC4 on July 1st, 1986.

